# A Systematic Review of Tobacco Industry Tactics in Southeast Asia: Lessons for Other Low- And MiddleIncome Regions

**DOI:** 10.34172/ijhpm.2020.97

**Published:** 2020-06-21

**Authors:** Gianna Gayle Herrera Amul, Grace Ping Ping Tan, Yvette van der Eijk

**Affiliations:** ^1^Lee Kuan Yew School of Public Policy, National University of Singapore, Singapore, Singapore.; ^2^Saw Swee Hock School of Public Health, National University of Singapore, Singapore, Singapore.

**Keywords:** Tobacco Industry Tactics, Transnational Tobacco, Southeast Asia, Tobacco Control, LMICs

## Abstract

**Background:** Transnational tobacco companies (TTCs) have a well-established presence in Southeast Asia and are now targeting other low- and middle-income countries (LMICs), especially Africa. While the tobacco industry’s tactics in Southeast Asia are well documented, no study has systematically reviewed these tactics to inform tobacco control policies and movements in Africa, where the tobacco epidemic is spreading.

**Methods:** We conducted a systematic literature review of articles that describe tobacco industry tactics in Southeast Asia, which includes Singapore, Indonesia, Malaysia, the Philippines, Myanmar, East Timor, Thailand, Cambodia, Vietnam, Laos, and Brunei. After screening 512 articles, we gathered and analysed data from 134 articles which met our final inclusion criteria.

**Results:** Tobacco transnationals gained dominance in Southeast Asian markets by positioning themselves as good corporate citizens with corporate social responsibility (CSR) initiatives, promoting the industry as a pillar of, and partner for, economic growth. Tobacco transnationals also formed strategic sectoral alliances and reinforced their political ties to delay the implementation of regulations and lobby for weaker tobacco control. Where governments resisted the transnationals’ attempts to enter a market, they used litigation and deceptive tactics including smuggling to pressure governments to open markets, and tarnished the reputation of public health organizations. The tobacco industry undermined tobacco advertising, promotion and sponsorship (TAPS) regulations through a broad range of direct and indirect marketing tactics.

**Conclusion:** The experience of Southeast Asia with tobacco transnationals show that, beyond highlighting the public health benefits, underscoring the economic benefits of tobacco control might be a more compelling argument for governments in LMICs to prioritise tobacco control. Given the tobacco industry’s widespread use of litigation, LMICs need more legal support and resources to counter industry litigations. LMICs should also prioritize measures to protect health policy from the vested interests of the tobacco industry, and to close regulatory loopholes in tobacco marketing restrictions.

## Background

In Southeast Asia, smoking rates are among the highest globally with an average male smoking prevalence of 42%.^1^ As of 2017, the region had 122 million adult smokers.^2^ Southeast Asia also remains a lucrative market for transnational tobacco companies (TTCs),^3^ which have operations in multiple countries to manufacture, distribute and market their products, often using aggressive marketing and lobbying tactics.

While Southeast Asia now has a well-established tobacco epidemic, TTCs have, in more recent decades, also pursued other low- and middle-income countries (LMICs).^4^ TTCs used sophisticated marketing tactics, smuggling, aggressive lobbying, and trade threats to open up and gain dominance in LMICs in Eastern Europe,^5-7^ Latin America,^8-10^ and the Middle East.^11^ As a result, TTCs managed to gain significant political and economic clout, influence policy to their advantage, and increase smoking rates in these regions. Consequently, smoking is no longer an epidemic restricted only to high-income countries (HICs). Eighty per cent of the world’s smokers now live in LMICs, where the burden of tobacco-related diseases hit the hardest by the substantial healthcare costs and lost human capital.^12^

Africa in particular holds much promise for tobacco transnationals. Amid ageing populations and slowing population growth in many parts of the world, including Southeast Asia,^13^ Africa’s population is growing,^14^ and by 2050 it is predicted that over 33% of the world’s youths will be in sub-Saharan Africa.^15^ Cigarette demand grew by 44% between 1990 and 2012 in 22 African nations representing 80% of the region’s population.^16^ While smoking prevalence is projected to decrease in most world regions, it is expected to increase from 12.8% to 18.1% in the African region by 2025 amid slow implementation and ineffective enforcement, industry interference, and inadequate resources for tobacco control.^17-19^

Although the tobacco epidemic started in ‘Western’ HICs,^20^ TTCs have adapted their tactics to target LMICs.^4^ LMICs generally have weak monitoring of tobacco use and policies, less resources to resist industry litigations, and are more susceptible to political corruption, trade pressure, exploitation of political instability and the industry’s promises of economic prosperity.^4,21-26^ TTCs’ tactics have also evolved since they dominated North American and West European markets in the 1930s, focusing increasingly on trade litigation and more innovative marketing strategies to circumvent advertising regulations.^4,27-30^

In preventing new tobacco epidemics in Africa, it is important to look to the experiences of other LMIC regions, such as Southeast Asia, where tobacco transnationals became established in more recent decades. Much like Africa now, Southeast Asia was a prime target for TTCs in the 1980s,^31,32^ when its population was growing with affluence,^33,34^ and when governments opened up to international trade in their pursuit of economic growth.^35^ While the tobacco industry’s tactics in Southeast Asia are well-documented, no study has systematically reviewed these tactics.

The aim of this study was to systematically review literature that describes tobacco industry tactics in Southeast Asian countries, to inform tobacco control policies and movements in other LMICs, especially in the African region, where the tobacco epidemic is spreading. We defined ‘tobacco industry tactic’ as any tactic, direct or indirect, to increase tobacco sales. We defined ‘Southeast Asia’ as the geographical region that includes Singapore, Indonesia, Malaysia, the Philippines, Myanmar, East Timor, Thailand, Cambodia, Vietnam, Laos, and Brunei.

## Methods

### Search Strategy

In March 2019, we searched databases for academic literature (eg, PubMed, Embase) and grey literature (eg, OAIster, Business Source Premier, publications of regional NGOs) using the search strings: “tobacco industry” AND (Asia OR ASEAN OR Singapore OR Indonesia OR Malaysia OR Philippines OR Myanmar OR Burma OR Timor OR Thailand OR Cambodia OR Vietnam OR Laos OR Brunei).

### Selection Criteria

**Figure 1:**
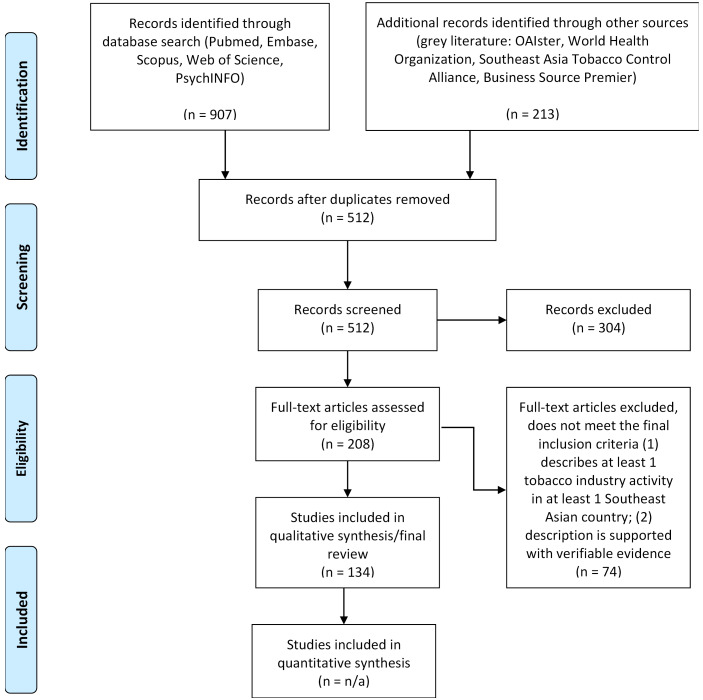
Study Selection Flowchart, Systematic Review of Tobacco Industry Tactics in Southeast Asia.

A total of 1120 articles were included for the initial abstract screening (Figure 1). 512 articles met our initial inclusion criteria: (1) available in English, (2) covers any of the Southeast Asian countries, and (3) mentions tobacco or tobacco industry. After reading the full text of these 512 articles, 134 articles from 1983 to 2019 met our final inclusion criteria for synthesis: (1) describes at least one tobacco industry activity in at least one Southeast Asian country; (2) activity is clearly described and supported with verifiable evidence. This systematic review followed the Preferred Reporting Items for Systematic Reviews and Meta-analysis (PRISMA) statement.

### Data Extraction

Data extraction was undertaken by the first and second authors using a standard template which included: (1) detailed information about tobacco industry strategy in Southeast Asia, and; (2) tagged references on countries covered, year or time period of the industry activity, tobacco industry tactic, arguments or issues (see Protocol, Supplementary file 1).

### Quality Assessment

The data extracted was then reviewed by the third author to check that all inclusion criteria were met and to agree on the categorization of industry tactics. All differences were discussed between all authors until agreement was reached.

### Data Analysis

**Figure 2:**
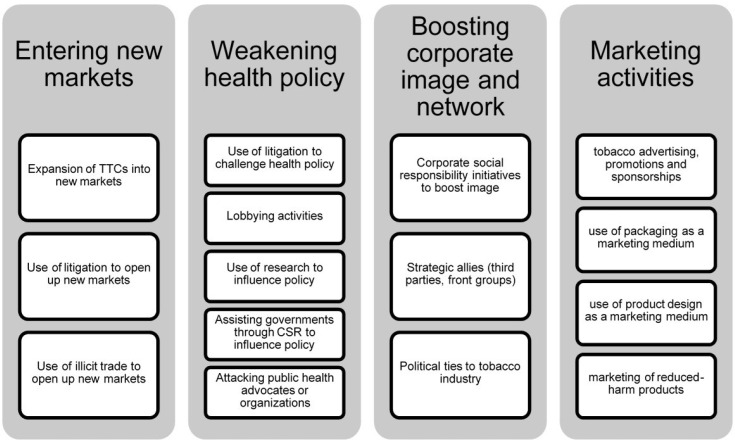
Tobacco Industry Tactics in Southeast Asia. Abbreviations: TTCs, transnational tobacco companies; CSR, corporate social responsibility.

We used an inductive coding method. We identified codes relating to tobacco industry activities based on our data, and subsequently refined and categorized these codes in an iterative process between all authors. Any discrepancies were resolved via discussions between all authors until agreement was reached. Thirteen subcategories relating to four broader themes emerged from our data (Figure 2). The data from the articles were then synthesized in a narrative.

## Results

### Entering New Markets

#### Expansion of Transnational Tobacco Companies Into New Markets

TTCs British American Tobacco (BAT), Imperial Tobacco, Philip Morris International (PMI) and Japan Tobacco International (JTI) started aggressively pursuing new Southeast Asian markets in the 1980s and 1990s, after tobacco consumption started dropping in HICs.^36,37^ In the countries with free port status (Singapore, the Philippines), TTCs already had well-established markets, while in other Southeast Asian countries, the industry started to dominate markets after the countries opened up trade. In Cambodia, Thailand and Vietnam, the tobacco industry was state-owned,^38^ while Indonesia, Myanmar, and the Philippines had well-established domestic tobacco industries before transnationals entered.^39-42^

TTCs penetrated Laos, Cambodia, Vietnam, Myanmar, Malaysia, the Philippines, and Indonesia through joint ventures, investments, mergers or licensing agreements with local tobacco companies, state-owned industries or governments. This gave TTCs financial and political leverage over tobacco control policies, led to domestic-transnational alliances to form a unified industry lobby, and provided them with direct links to trade, industry and finance ministries (Table 1).^36,43-47^

**Table 1:** Joint Ventures, Investments, Mergers and Licensing Agreements of Tobacco Transnationals in Southeast Asia

**Country**	**Transnational**	**Local Entity**	**Details**	**Source**
Laos	Imperial Brands (formerly Imperial Tobacco)	Lao Government	Joint venture to form Lao Tobacco Ltd through Coralma Intl, S3T	^2^
Cambodia	BAT	Cambodian Tobacco Company	Joint venture in 1996 between BAT-Singapura United Trading and Cambodian Tobacco Company (Kong Triv)	^49^
Vietnam	BAT	Vinataba	Joint venture in 2001	^45^
Myanmar	BAT	Union of Myanmar Economic Holdings	Joint venture between Rothmans of Pall Mall Myanmar and Union of Myanmar Economic Holdings (military regime’s economic arm) until 2004	^38^
	BAT	IMU (Sein Wut Hmon Group)	Majority stake ($50m) in joint venture in 2014	^50^
Philippines	RJ Reynolds	Fortune Tobacco Company	Licensing agreement in 1974	^42^
	PMI	La Suerte Cigar and Cigarette Company Fortune Tobacco Company	Licensing agreement in 1955-2002 Merger to form PMFTC, $20m investment in 2010	^2,42,51^
	JTI	Mighty Corporation	$936m investment in 2017	^2^
Indonesia	PMI	Sampoerna	$5.2b investment (98% share) in 2005	^2,46,52^
	BAT	Bentoel	Acquisition for $494m (99.7% share) in 2009	^2,51,52^
	JTI	Karyadibya Mahardhika, Surya Mustika Nusantara	Acquisition for $677b in 2017	^2^
Malaysia	PMI	Philip Morris (Malaysia) Sdn Bhd	$40m investment in 2005	^51^

Abbreviations: BAT, British American Tobacco; PMI, Philip Morris International; JTI, Japan Tobacco International.

Notably, the agreement between Imperial and the Lao Government’s Lao Tobacco Ltd^2^ resulted in a tax agreement which barred the Lao government from increasing tobacco taxes from 2001 to 2026.^38,48^ The joint venture between BAT and Vinataba, Vietnam’s state-owned tobacco industry, gave BAT direct links to the Ministry of Trade and Industry and Ministry of Finance.^44,45^

In Indonesia, where transnationals faced strong competition from domestic kretek manufacturers,^46^ transnationals leveraged on the kretek industry’s political influence by forming lobby alliances with them to counter regulations.^47^ A decade later, after the transnationals had gained more political influence, they started acquiring domestic industry shares.

#### Use of Litigation to Open up New Markets

Thailand, which had a government-owned tobacco monopoly (Thailand Tobacco Monopoly (TTM)) since 1939, was resistant to TTCs.^53^ TTCs responded with litigation against Thailand and lobbying through an industry alliance (US Cigarette Export Association) with the US Trade Representative, Thailand’s Ministry of Finance and Ministry of Commerce, pro-entry politicians and Thai exporters to open up the tobacco market using Section 301 of the 1974 US Trade Act.^54^ In 1989, the US Trade Representative referred its dispute to the World Trade Organization and in 1990, forced Thailand to lift its tobacco import restrictions.^32,36,55,56^ TTCs then ‘localized’ by forming alliances and business relationships with politically influential Thais.^56^

#### Use of Illicit Trade to Open up New Markets

BAT engaged in illicit trade operations while negotiating a joint venture with Vinataba, Vietnam’s state-owned tobacco industry, to create local demand and leverage access to the market after Vietnam’s import ban on cigarettes in 1990.^45,49^ BAT’s joint venture in Cambodia was crucial for its smuggling operations into Vietnam, due to its proximity and manufacturing facilities.^49,57,58^ After Vietnam’s market opened up, BAT controlled the prices of locally produced and smuggled cigarettes to aid the transition from illicit trade to legal sales.^45,57^ BAT also smuggled its brands into Laos, Myanmar, Thailand, Malaysia, Singapore, and the Philippines,^57,59,60^ and used its Singapore-based distribution partner, Singapura United Tobacco Limited, to oversee smuggling in Southeast Asia.^57,61^ BAT also used legal trade flows as cover for its smuggling operations in the region, blurring the line between legal and illegal operations through ‘partial duty paid’ products and ‘duty free’ leakage.^57^

### Weakening Health Policy

#### Use of Litigation to Challenge Health Policy

TTCs launched legal challenges against governments based on national laws, trade law and international agreements to undermine health policies and intimidate governments, both at the national and local levels, in Southeast Asia (Table 2).^62,63^ There were 39 such legal challenges in Indonesia, Malaysia, the Philippines, and Thailand documented in the literature. While most of these lawsuits were against national governments, the tobacco industry also used litigation to intimidate local governments such as Balanga, a small city in the Philippines.

**Table 2:** Tobacco Industry-Led Lawsuits Against Governments in Southeast Asia

**Country**	**Number/Type of Lawsuit**	**Details**	**Sources**
Indonesia	13 vs. National government	To challenge Health Law 36/2009 on addictive nature of cigarettes, graphic health warnings, smokefree legislation	^2,64,65^
	1 vs. Jakarta	To challenge smokefree legislation	^2^
	1 vs. Bogor	To challenge smokefree legislation	^2^
Malaysia	3 vs. Health Ministry	To challenge price increases, requirement of Health Ministry approval for cigarette pricing	^2,64^
Philippines	11 vs. National government	To challenge graphic health warnings, authority to regulate tobacco products, marketing restrictions	^2,63,64,66^
	2 vs. Balanga	To challenge the Tobacco-Free Generation law and other sale restrictions	^67^
Thailand	8 vs. National government	To challenge graphic health warnings and other tobacco control measures	^2,62,64^

#### Lobbying Activities

Tobacco industry front groups and alliances argued that the industry contributed to economic development and employment in the region, especially for tobacco farmers.^49,52,66,68-71^ The industry formed alliances to strengthen their lobby,^47,52,66,72,73^ which typically consisted of TTCs and domestic tobacco companies, the media, tobacco farmers, local governments, ministries, and influential locals.^47,56,73,74^ These alliances resulted in weaker marketing regulations,^66^ or where regulations could not be avoided, delays in their implementation.^62-64,70,75-77^

In Indonesia, tobacco lobbyists successfully blocked Indonesia’s accession to the World Health Organization (WHO Framework Convention on Tobacco Control (FCTC).^47,52,70^ Through Indonesian associations, tobacco companies lobbied for the Indonesian government’s ‘Roadmap of Tobacco Products Industry and Excise Policy 2007-2020,’ which called for a 12% increase in annual cigarette production up to 2020 and tobacco industry participation in policy-making.^52^ In 2009, industry front groups lobbied against a proposed clause in the Indonesian National Health Bill that would identify tobacco as an addictive substance, which was later removed in the short period after the bill was passed by Parliament and before the President signed the bill into law.^73,78^

The majority of TCCs’ lobbying documented in Southeast Asia were against tobacco taxes and graphic health warnings. TTCs blocked or weakened tobacco taxes by publishing resource manuals not aligned with FCTC guidelines,^79-81^ lobbying for ad valorem taxes,^82^ meeting legislators,^68^ mounting media campaigns,^83^ and bribing government officials.^42^ TTCs spent decades lobbying against graphic health warnings in Singapore,^63^ Malaysia,^69^ Cambodia,^63^ and the Philippines.^42^ In Malaysia, these efforts resulted in weaker regulations from 1970 to 1995.^69^ In the Philippines, the tobacco industry was able to delay the implementation of graphic health warnings for three decades.^42^

#### Use of Research to Influence Policy

TTCs also used researchers in Southeast Asia to substantiate their lobby, particularly against smokefree legislation and tobacco taxes (Table 3). TTCs launched initiatives like the International Environmental Tobacco Smoke Consultants Program (1989-1999) and the Asian Regional Tobacco Industry Science Team (1996) to counter smoke free measures by obscuring the effects of secondhand smoke compared to outdoor pollution and promoting ineffective ventilation technologies.^84-86^ TTCs recruited consultant researchers from Thailand, Indonesia, Malaysia, Singapore and the Philippines and were compensated with consultancy fees via Covington and Burling, a Washington DC-based law firm.^85^ TTCs encouraged the consultants to solicit research assignments from government bodies to boost the group’s credibility, and amplified their publicity with media tours, editorial columns, and national and international conferences.^42^ The consultants attended at least 34 conferences from 1988 to 1990 and developed a close relationship with the Asian Association of Occupational Health.^84^

**Table 3:** Researchers in Southeast Asia Funded by the Tobacco Industry^42,84,87^

**Country**	**Name**	**Research Area or Affiliation at Time of Involvement**
Philippines	Ben Ferer	Architecture
	Luis M. Ferrer	Director of Health Infrastructure Service, Ministry of Health
	Benito Reverente	Occupational Health/Pulmonary Medicine; Member of WHO Occupational Health Panel
	Camilo Roa Jr	Pulmonary medicine
	Lina Somera	Occupational health and biostatistics; Head of Public Health at University of the Philippines
	Domingo Aviado	Pharmacology
	Daniel A Witt	President, International Tax and Investment Center (Philippines, Thailand, Myanmar)
Singapore	Choong Nam Ong	Occupational Hygienist, National University Hospital
	Adrian Cooper	CEO, Oxford Economics (Singapore office)
Malaysia	Krishna Ramphal	Department of Community Medicine, National University of Malaysia
Thailand	Malinee Wongphanich	President, Asian Association for Occupational Health
	Mathuros Ruchirawat	Vice President for Research, Chulabhorn Research Institute; Associate Professor of Pharmacology, Mahidol University

Abbreviations: WHO, World Health Organization; CEO, chief executive officer.

PMI took advantage of its links with scientists at Thailand’s Chulabhorn Research Institute, a WHO Collaborating Centre with ties to the Thai Royal family, to challenge Thailand’s smokefree legislation and the 1992 Tobacco Products Control Act.^56,87^ Moreover, tobacco transnationals co-opted scientists from various disciplines, some of whom were unaware of the tobacco industry associations, to lobby against smokefree legislations.^56,87^ PMI also wielded its ties with the Philippines’ Department of Science and Technology to offer research funds to a university teaching hospital in Bicol, Philippines, but was blocked by the Department of Health.^88,89^

Through industry-funded research, TTCs lobbied against tobacco taxes in Southeast Asia using illicit trade as an argument.^42,45,56,61,70,80,82,90-92^ An industry-funded report by the International Tax and Investment Center (ITIC) and Oxford Economics, the “Asia-14 Illicit Tobacco Indicator 2013,” overestimated illicit trade in the region and advised governments to avoid ‘excessive’ taxation to prevent illicit trade.^76,79-82,91,93^ ITIC was funded by tobacco companies and Board of Directors included members from PMI, Imperial, JTI, and BAT.^82^ The report’s authors had also previously been employed in the tobacco industry.^82^ TTCs ensured that the report received widespread media coverage in the region,^82,91^ and used it to lobby against tobacco taxes^61,82^ and to offer ‘technical assistance’ to governments on excise tax reform.^76,79,81,93^

#### Assisting Governments Through Corporate Social Responsibility to Influence Policy

TTCs set up anti-smuggling corporate social responsibility (CSR) initiatives to counter tobacco taxes in Southeast Asia, in the form of training and assistance to governments in anti-smuggling raids,^45,64,68,76,77,80,93-95^ contributions to national and international anti-smuggling agencies including Interpol,^45,64,70,77,81,93^ and partnerships with governments, trade and industry ministries, justice ministries and customs departments on anti-smuggling issues in the Philippines, Thailand, Vietnam, Cambodia, and Malaysia.^45,68,76,80,81,93,94,96^ TTCs’ collaboration with Vietnam’s Ministry of Trade and Industry resulted in 50% of Vietnam’s tobacco control fund being allocated to control smuggling.^77,95^ TTCs also proposed industry-developed track and trace systems to governments, particularly Codentify, a ‘digital tax verification’ system to replace tax stamps and fiscal markers.^97^

TTCs promoted voluntary regulations in Southeast Asia to dilute regulations on tobacco marketing and ingredient disclosure,^50,71^ and to supersede the FCTC.^98^ TTCs also promoted their own CSR programmes to prevent youth smoking in Thailand, Malaysia and the Philippines to improve their image and provide opportunities for engagement with retailers and government officials.^69,71,88,98-100^ TTCs espoused the concept of ‘smoker accommodation’ to undermine smokefree laws, by donating ashtrays to public places,^101^ and by funding the construction of airport smoking rooms.^94^

#### Attacking Public Health Advocates or Organisations

TTCs systematically identified public health advocates and health promotion organisations in Southeast Asia, monitored their publications and finances, and in some cases, attacked their reputation to limit their influence and credibility.^102^ In Indonesia, TTCs set up a billboard with the names and photos of public health advocates, branding them as ‘enemies,’^65^ and in Thailand, TTCs pressured the Thai government to investigate and restructure the Thai Health Promotion Board.^76,81,93^

### Boosting Corporate Image and Network

#### Corporate Social Responsibility Initiatives to Boost Image

From 2010 to 2018, TTCs spent over $80 million on CSR initiatives in Southeast Asia,^2,103,104^ much of which was spent on promoting these initiatives to boost corporate image.^105^ These CSR initiatives were either funded directly or channelled through foundations or industry-related associations (Table 4), and usually focused on human rights, the environment, employee welfare, education, social welfare, poverty, disaster relief, or arts and culture.^2,42,68,71,74,77,88,89,93,99,103-111^ TTCs used these initiatives to gain publicity,^110^ promote their products,^99,112^ enhance their network and image with governments,^76,77,81,95,105,111,113^ local organizations,^110^ and international organizations (UNDP, ILO, UNICEF),^103,110,114^ and to engage tobacco farmers in pro-tobacco lobbying.^71,106,108,115,116^

**Table 4:** Foundations or Associations Used to Conduct CSR Activities in Southeast Asia

**Foundation or Association**	**CSR Activities**	**Sources**
BAT Malaysia Foundation	Scholarships, training, schools, community development	^52,88,110,112,116^
JTI Foundation	Disaster relief, prevention of human trafficking	^110,117^
KT&G Social Welfare Foundation	Disaster relief	^110,117^
Fundacion Altadis (Imperial)	Poverty relief, food security, environmental sustainability	^110,118^
American Chamber of Commerce Foundations	Education, poverty relief, disaster relief, environment	^81,110,119^
Jaime Ongpin Foundation	Environment, community development	^110,120,121^
Putera Sampoerna Foundation	Scholarships, training, schools, community development	^52,88,110,112,116^
Philip Morris Arts Foundation Thailand	ASEAN Art Awards	^88^
Tan Yan Kee Foundation (Fortune)	Not specified	^110^
Wong Chu King Foundation (Mighty)	Religious activities	^93,110^
Djarum Foundation	Not specified	^110^
KT&G Sunny Korea Welfare Foundation	Child welfare	^117^
PMI Thailand Population and Community Development Association	Scholarships, training, community development	^110^
PMI Thailand Chiangrai and Phayao Tobacco Curer, Planter and Seller Association	Scholarships, training, community development	^110^
PMI Thai Tobacco Growers, Curers and Dealers Association	Scholarships, training, community development	^107,110^
Eliminating Child Labour in Growing Tobacco Foundation	Child labour prevention	^103,114^

Abbreviations: BAT, British American Tobacco; PMI, Philip Morris International; JTI, Japan Tobacco International; CSR, corporate social responsibility.

TTCs regularly conducted CSR activities for tobacco workers but failed to improve their lives and well-being. In Cambodia, Indonesia and the Philippines, tobacco farmers entered into loan agreements with tobacco companies, trapping them into debt cycles and forcing them to sell tobacco leaf at a price set by manufacturers.^90,122^ In Malaysia, despite tobacco industry assistance and subsidies, tobacco farmers still bore the cost of tackling crop diseases and adverse weather conditions.^115^ In Vietnam, tobacco farmers were found to be earning less or only marginally more than non-tobacco farmers, and had more health complaints.^123^ In Indonesia, PMI subsidiary Sampoerna ignored complaints of mistreatment, low pay, poor living conditions, and unsafe working environments from its factory workers.^116^ Moreover, TTCs also profited from child labour in Southeast Asia,^103,105,110^ while their CSR initiatives to combat child labour in the region had little impact.^103,114^

#### Strategic Allies (Third Parties, Front Groups)

TTCs funded or partnered with front groups and third parties in Southeast Asia to gain access to governments, undermine tobacco regulations and boost their corporate image (Table 5).

**Table 5:** Front Groups and Third Parties Documented to Have Received Tobacco Industry Funding for Operations in Southeast Asia

**Organization**	**Sources**
**Formal business networks**	
International Business Chamber Cambodia	^68^
Cambodia Chamber of Commerce	^68^
Cambodian Federation of Employers and Business Associations	^68^
American Chamber of Commerce	^77,80,81,95,119^
**Associations/alliances**	
Gabungan Pengusaha Pabrik Rokok Indonesia (kretek manufacturers association)	^52^
Gabungan Produsen Rokok Putih Indonesia (white cigarette manufacturers association)	^52 65^
Alliance of Indonesia Tobacco Society	^65^
Indonesia Tobacco Society Movement (Gemati)	^65^
Central Java Regional Representatives Council	^83^
District Legislative Councils Association	^83^
Indonesian Tobacco Farmers’ Association	^83^
International Tobacco Growers Association	^2,64,124,125^
Thai Tobacco Trade Association	^70^
Tobacco Retailer Association (Thailand)	^126^
Malaysia Coffee Shop Association	^73^
Malaysia-Singapore Coffee Shop Proprietors General Association	^73,127^
**International agencies**	
International Union of Food	^103^
International Labour Organization	^103,73^
UNICEF	^103,73^
Interpol	^70,97^
**Think tanks**	
Institute for Democracy and Economic Affairs	^77^
International Tax and Investment Center	^76,81,93,128^

Abbreviation: UNICEF, The United Nations Children’s Fund.

#### Political Ties to Tobacco Industry

TTCs forged extensive ties with political and business elites in Southeast Asia (Table 6), especially in Indonesia, the Philippines,^42^ Cambodia,^63,68^ and Malaysia.^115^

**Table 6:** Political and Business Elites in Southeast Asia Reported to Have Conflicts of Interest With the Tobacco Industry

**Individual**	**Conflict of Interest**	**Sources**
**Indonesia**	
President Suharto’s brother	Joint venture with Gudang Garam founder Surya Wonowidjojo	^47^
President Suharto’s youngest son	Founded BPCC (Clove Support and Trading Board) clove monopoly in 1990	^47,113,129,130,131^
President Suharto’s cousin	Partnership with Australian Rothmans Holdings Ltd	^113,129^
President Suharto’s second son; owner of Indovision	Star TV Indonesia, which runs Indovision, owned by Phillip Morris director Rupert Murdoch	^113,129^
Raden Bagus Permana Agung Dradjattun, Expert for Ministry of Finance	Independent Commissioner of Sampoerna	^76,77,80,81,95^
Former Director-General of Customs and Excise	Commissioner of Sampoerna	^76,77,80,81,95^
Eddy Abdurrahman, Former Director-General of Customs and Excise in Ministry of Finance	BAT/Bentoel Independent Commissioner on BAT’s Board and Chairman of BAT/Bentoel Audit Committee	^76,77,80,81,95^
**Philippines**	
Estelito Mendoza, Former Solicitor General	Legal counsel to Lucio Tan (Chairman of Fortune Tobacco)	^81^
Edilberto Adan, Former Military Official	Director and President of Mighty Corporation	^81^
Oscar Barrientos, Former Judge	Executive Vice President of Mighty Corporation	^81^
Members of the Inter-Agency Committee on Tobacco	Employed in Philippine Tobacco Institute	^80,81,96^
**Cambodia**	
Senator	Chairman of BAT Cambodia	^77^
**Laos**	
Officials in Ministry of Finance	Members of the Board Management of Lao Tobacco Ltd	^81^
Officials in Ministry of Industry and Commerce	Members of the Board Management of Lao Tobacco Ltd	^81^
Retired Vice Director General of Enterprise Department	Board member of Lao Tobacco Ltd	^81^
**Malaysia**	
Tan Sri Datuk Dr Rebecca Fatima Sta Maria, Secretary-General of the Ministry of International Trade and Industry	Council member of Institute for Democracy and Economic Affairs, a tobacco industry-funded think tank	^77^
Tan Sri Datuk Abu Talib bin Othman, Former Attorney General	Chairman of BAT Malaysia	^94-96,115^
Tan Sri Dato’ Seri Dr Aseh, Former Secretary General of the Ministry of Home Affairs	Chairman of BAT Malaysia	^94-96^
Hon Dato’ Sri Mohamed Khalid Bin Hj Yusef, former Director General of Customs	Senior Adviser to ITIC	^80^
Tunku Tan Sri Mohamed bin Tunku Besar Burhanuddin, Former Chief Secretary	First chairman of Rothmans of Pall Mall (later BAT Malaysia)	^115^
Brother of Prime Minister, Dato’ Sri Mohamed Najib Abdul Razak	Non-executive director on BAT Malaysia’s board	^115^
Datuk Mohamad bin Fateh Din, Malaysian Communications and Multimedia Commission	Chairman of BAT Malaysia	^115^
Datuk Zainun Aishah binti, Malaysian Industrial Development Agency	Board member of BAT Malaysia	^115^
Tan Sri Kamarul bin Mohamed Yassin, Former Senator	Board member of BAT Malaysia	^115^
**Thailand**	
Permanent Secretary in the Ministry of Interior	TTM board member	^76,81,94,96^
Minister in the Prime Minister’s Office	Owner of a local tobacco leaf business	^76,81,94,96^
Former Lieutenant General	TTM Chairman	^76^
**Vietnam**	
Ha Quang Hoa, Former Deputy Director in Ministry of Trade and Industry	Vice Director at VINATABA	^81^
Vice Director in Ministry of Trade and Industry	Board member at VINATABA	^81^
Vu Van Cuong, Senior Member of Communist Party	Chairman of the Board of VINATABA	^81^
Ha Quang Hoa, Former Deputy Director in Ministry of Trade and Industry	Vice Director at VINATABA	^81^

Abbreviations: BPCC, Badan Penyangga dan Pemasaran Cenkgeh; BAT, British American Tobacco; ITIC, International Tax and Investment Center; TTM, Thailand Tobacco Monopoly.

### Marketing Activities

#### Tobacco Advertising, Promotions and Sponsorships

TTCs’ marketing tactics varied widely in Southeast Asia, depending on the strength and enforcement of regulations on tobacco advertising, promotion and sponsorship (TAPS).

In Indonesia, TTCs exploited the lack of marketing restrictions with heavy direct, outdoor, and point-of-sale advertising.^64,124,132^ In restricted markets that still allowed point-of-sale advertising (eg, the Philippines, Vietnam), TTCs invested heavily in point-of-sale advertising, including entire shops, buildings and cigarette booths painted with brand colours, illuminated brand advertisements and branded parasols, and the use of promotional girls to sell cigarettes.^89,109,132-139^ In countries with weakly enforced marketing restrictions, TTCs violated marketing regulations with illegal installation of point-of-sale displays, vending machines, and promotional items. TTCs encouraged retailers to violate regulations through regular engagements, including frequent visits, free merchandise, rewards for meeting sales targets, and by hosting annual dinners and musical events for retailers.^64,111,124,127,132,135,140^ After Thailand’s tobacco display ban, TTCs circumvented restrictions with mobile cigarette shops and transparent point-of-sale displays showing brand logos and colours.^126^

TTCs utilized trademark diversification^50,56,99,141-149^ by using tobacco brands on non-tobacco products such as lighters,^143^ wine coolers,^142^ luxury products, clothing, or travel agencies and holiday themes.^99,148,149^ Tobacco companies also operated trademark diversification businesses, notably in Malaysia, including BAT’s ‘World Investment Company’ (Benson and Hedges Bistros, Kent Travel), Brown and Williamson’s ‘Diversification International Products’ (Kent Leisure Holidays), PMI’s ‘International Trademark’ (Marlboro Classics) and R.J. Reynolds’ ‘Worldwide Brands’ (Camel Stores).^141^

TTCs also invested heavily in sponsorship of popular sports, music concerts, movies, fashion events and the arts, to associate their products with youth, popular culture, adventure and leisure. Notable arts sponsorships in the region include Philip Morris’ ASEAN Art Awards^150^ and Sampoerna’s SoundrenAline concerts in Indonesia.^151^ In Indonesia, which has no restrictions on tobacco industry sponsorship, an estimated 85% of Indonesian youths had attended at least one tobacco-sponsored event in 2007.^52^ TTCs also sponsored professional football in Vietnam,^136^ the Philippines’ annual Marlboro Tour (cycling),^152^ and Malaysia’s Formula 1 Grand Prix,^148,153^ and 2002 FIFA World Cup.^154^ Sports sponsorships functioned as cross-border advertising to reach countries with stricter advertising bans (Thailand, Singapore).^111,155,156^ Sports sponsorships also resulted in the delay of some tobacco control measures, such as Malaysia’s 5-year freeze on tobacco taxes in exchange for BAT’s sponsorship of the 1998 Commonwealth Games.^73,141^

More recently, TTCs have used social media platforms (eg, Facebook, Flickr, Instagram) to promote their brands and sponsored events in Southeast Asia.^157^ Despite Malaysia’s ban on internet advertising, BAT, PMI and JTI have all used social media, particularly Facebook, to promote their products.^64,124^ Sampoerna used Instagram to promote its SoundrenAline concerts with brand hashtags, and used its ‘Go Ahead People’ art website to collect user data for marketing through social media account registration, promotions, and creative opportunities in music, fashion, photography and arts.^151^

#### Use of Packaging as a Marketing Medium

Tobacco companies used pack colours and designs to differentiate brand variants, create a false impression of reduced harm, and replace banned descriptors.^134^ Companies have used a variety of innovative pack designs to promote their brands in Southeast Asia, such as wallet packs, twin- or multi-packs, promotional packs, expanded size packs, lipstick packs,^89,134,158,159^ glow in the dark packs, transparent packs,^142^ and packs with rounded corners.^160,161^ Tobacco companies also used pack designs to dilute or cover up graphic health warnings.^89,99,134,159,162^ In some countries, tobacco companies did not comply to graphic health warnings,^65,77,162,163^ and misled retailers about the enforcement start date to delay impact of the graphic health warning regulations.^162^

To encourage youth smoking in Southeast Asia, tobacco companies sold cigarettes in smaller packs of 5,^99,158,164^ 10, and 14.^99,142^ The sale of single sticks, sometimes illegally, was also reported to be common in the region.^90,99,158^

#### Use of Product Design as a Marketing Medium

Tobacco companies carefully researched Southeast Asian youth to develop products to appeal to them,^42,100,147^ notably cigarettes with novelties such as added flavours, flavour capsules, and product design novelties (sweetened tips, a dial to control menthol delivery).^89,100,117,147,161^ To target health-conscious people in Malaysia and Singapore, tobacco companies marketed their imported menthol, ‘light’ and ‘mild’ brands with imagery, colours and messages that conveyed a lower health risk, despite knowing that these products were just as harmful as regular brands.^47,90,142,147^ Companies also used filters, such as charcoal filters or white-coloured filters, to convey a lower health risk, and used descriptors alluding to cutting-edge technology (eg, ‘Triple Filter Charcoal’) when descriptors such as ‘light’ and ‘mild’ were banned.^161^ Tobacco companies also targeted women in the region with light, menthol-flavoured and ‘slim’ brands.^42,52,117,130,138,147,150,165^

#### Marketing of Reduced-Harm Products

Tobacco companies have aggressively marketed e-cigarettes in Malaysia, Thailand and the Philippines with billboards, point of sale advertising, promotions, and online or temporary pop-up shops. E-liquids have been sold in a wide variety of flavours alongside other consumer products. Companies have provided little, if any information on the ingredients inside e-liquids or their safety, yet claim they are a safe alternative to tobacco.^166^

## Discussion

Our findings show that, in Southeast Asia, TTCs used arguments of economic growth and developed a good corporate image through CSR to forge ties with the local industry, policy-makers, researchers, political and economic elites, and key organizations to enter new markets and weaken tobacco policies. Where countries resisted, TTCs resorted to litigation and illicit trade. In countries with strong tobacco control movements, TTCs engaged in ‘smear’ campaigns against public health organizations. Our findings also highlight TTCs’ heavy use of point of sale advertising, sponsorships, social media and packaging to get around regulatory loopholes and create demand for their products in Southeast Asia, especially among youth.

As in Southeast Asia, TTCs regularly conduct CSR initiatives in Africa focusing on good employment practices, the environment, child welfare,^167^ education, disaster relief, arts and culture,^168^ and business development,^169^ and provide ‘technical assistance’ on anti-smuggling measures to African governments.^167^ TTCs have also provided funding to universities in South Africa.^168,170,171^ In their lobbying efforts, TTCs use arguments of economic growth and good corporate citizenship as documented in Nigeria^172,173^ and South Africa.^174^ Our findings indicate that these activities are not intended to aid countries and unlikely to have a meaningful impact on their economic or social welfare. Rather they are a means to improve corporate image, gain access to policy-makers, lobby against health policies and gain dominance in new markets.

The experiences of Southeast Asia highlight the importance of protecting health policy in LMICs from the vested interests of the tobacco industry, in line with FCTC Article 5.3.^175^ This is also evidenced in the contrasting examples of Singapore and Indonesia. Singapore, which follows strict internal policies in line with Article 5.3,^2^ has the region’s most comprehensive tobacco policy and a daily smoking prevalence of 12.0%.^176^ In contrast, Indonesia’s policy-makers have extensive ties with tobacco companies, and Indonesia has the region’s highest smoking rates^177^ and weakest tobacco control measures.^2^ The case of Indonesia also highlights the power of economic arguments, suggesting that arguments which emphasise the economic costs of the burden of tobacco, and benefits of tobacco control to economies and sustainable development, might be more persuasive to governments when pushing for tobacco control in LMICs.^178,179^

Our study highlights the tobacco industry’s use of litigations to challenge tobacco control measures of Southeast Asian governments, both at the national and local level. This is consistent with other studies that illustrate the tobacco industry’s increasing reliance on litigation as an intimidation tactic (regulatory chilling) to dissuade governments, especially those with limited resources, from implementing health policies.^4^ In the past decade, TTCs have threatened litigations against LMICs in Africa including Congo, Burkina Faso, Ethiopia, Togo, Gabon and Namibia,^180,181^ and filed lawsuits, all of which TTCs lost, against governments in South Africa, Kenya and Uganda over their tobacco control laws.^182^ Although the industry’s legal cases are often weak, and ultimately lost in courts, the costs of defending against these legal challenges can intimidate LMICs into lifting restrictions.^183,184^ More resources to fight against tobacco industry litigations should be made available to LMICs.

Southeast Asia also illustrates the vast extent to which TTCs circumvent weak or poorly enforced TAPS restrictions with point of sale advertising, sponsorships, trademark diversification strategies, packaging, and the sale of ‘mini packs’ and single sticks to target youth. In other LMICs with partial or poorly enforced TAPS restrictions, similar tactics have been observed.^185,186^ In Southeast Asia, only Thailand and Singapore have restricted all forms of direct and indirect TAPS, and have committed to plain packaging.^187^ More Southeast Asian countries, and other LMICs, need to close the existing loopholes in their TAPS regulations.

## Limitations

This study was based on published information of tobacco industry activities; thus, information may be limited in countries with less freedom of information, or where the topic has not been the subject of local research or media unless raised by regional or international civil society organisations. Our study included only English-language articles, although very few (less than five) were excluded based on this criterion. While the study offers a review of what is known about the tobacco industry’s tactics in Southeast Asia, the study does not delve deeper into what the industry has done beyond what is already published, or tobacco industry tactics in other regional, cultural or political contexts. Moreover, while East Timor was included in the study, we were not able to find studies on TTCs’ tactics in East Timor.

## Conclusion

The tobacco industry’s tactics in Southeast Asia are being replicated in other LMIC regions, especially Africa. Tobacco business is detrimental to economies, yet in Southeast Asia, TTCs have used economic arguments to promote the tobacco industry and weaken tobacco control policies. Our findings suggest that policy-makers in LMICs need to recognize the burden of tobacco-related diseases on public health and the economy and institutionalize strong mechanisms to protect health policies from the vested interests of the tobacco industry. Policy-makers in LMICs also need to pay attention to civil society organizations that monitor the tobacco industry which help build the evidence of the tobacco industry’s tactics to weaken tobacco control measures. Policy-makers and tobacco control movements in LMICs also need better resources to fight industry counter-lobbying and litigation and should prioritise implementing FCTC-compliant regulations on all forms of tobacco marketing.

## Ethical issues

Not applicable.

## Competing interests

The authors declare that they have no competing interests.

## Disclaimers

The views of the authors in the study do not reflect the position of the institutions they are affiliated with.

## Authors’ contributions

YV conceptualized and designed the protocol for the study. YV collected the initial list of references for systematic review. GGHA and GPPT acquired, analysed and interpreted the data. All authors contributed in the drafting and revising the manuscript and were all involved in the final approval of the version to be published. All authors agree to be accountable for all aspects of the study.

## Authors’ affiliations

^1^Lee Kuan Yew School of Public Policy, National University of Singapore, Singapore, Singapore. ^2^Saw Swee Hock School of Public Health, National University of Singapore, Singapore, Singapore.

## Supplementary files

Supplementary file 1. Protocol.
