# Supplementary material for: A Systematic Review of Tobacco Industry Tactics in Southeast Asia: Lessons for Other Low- And MiddleIncome Regions
Source: Int J Health Policy Manag. 2020 Jun 21;10(6):324–37. doi: 10.34172/ijhpm.2020.97 (PMC9056152; doi:10.34172/ijhpm.2020.97)
Supplement: Supplementary file 1 — Protocol. [file ijhpm-10-324-s001.pdf]

Supplementary file 1. Protocol

AIM & SCOPE

To find out what is currently known about tobacco industry activities in Southeast Asia. Southeast Asia is defined as the geographical region that includes Singapore, Indonesia, Malaysia, the Philippines, Myanmar (formerly known as Burma), East Timor, Thailand, Cambodia, Vietnam, Laos, and Brunei. All of these, except East Timor, are also members of the Association of Southeast Asian Nations (ASEAN).

Protocol

Data gathering and screening

(1) Search databases using specific search string.
(2) Title/abstract screen: add references that meet inclusion criteria to EndNote file 'screen 1.'
(3) Remove duplicate EndNote files from 'screen 1.'
(4) Full text screen: read full text of all references in 'screen 1.'
(5) Add those that meet inclusion criteria to EndNote file 'screen 2.'
(6) Data analysis: analyze full text for all references in 'screen 2.'

Search string:
"tobacco industry" AND (Asia OR ASEAN OR Singapore OR Indonesia OR Malaysia OR Philippines OR Myanmar OR Burma OR Timor OR Thailand OR Cambodia OR Vietnam OR Laos OR Brunei)

Inclusion/Exclusion Criteria	Include	Action
Title/abstract screening:<br>1. Is the study in English, or is there a high quality English translation available?<br>2. Does it potentially cover any of the Southeast Asian countries?<br>3. Does it mention tobacco or tobacco industry?	If all yes	Import to EndNote file #1

Full text screening:<br>1. Is the paper written in English, or is there a high quality English translation available?<br>2. Does the study describe at least one tobacco industry activity in at least one Southeast Asian country?<br>3. Is this activity clearly described and supported with verifiable evidence (eg, a reference or photograph)?	If all yes	Copy to EndNote file #2 for data analysis

Results of first screening (title and abstract):

**Database**	**Search dates**	**Total hits**	**Total to EndNote**
PubMed	5/3/2019	553	211
Embase	5/3/2019	680	269
Scopus	5/3/2019	241	194
Web of Science	5/3/2019	99	85
PsychINFO	5/3/2019	7,965	148
OAIster	8/3/2019	147	42
WHO publications	7/3/2019	76	0
SEATCA publications	7/3/2019	78	54
UCSF bibliography	6/3/2019	29	22
Business Source Premier	6/3/2019	137	95
		TOTAL	1,120
**TOTAL (ALL DUPLICATES REMOVED)**			**512**

Results of second screening (full text):

- 134 articles met final inclusion criteria.
- Date range: 1983-2019.

Data extraction

Specific instructions for data extraction:

1. Record in detail any information about tobacco industry strategies in Southeast Asian countries. Draw out quotes where relevant, otherwise paraphrase. Stick to a bullet point format.
2. Do NOT record information about industry activity in countries outside Southeast Asia. Do NOT record anything on Southeast Asia tobacco policy, tobacco issues, etc unless it is directly related to industry activity.
3. As you go, assign tags to each reference. These are like keywords on what the reference covers. Tags should include (1) the countries covered, (2) year or time period in which industry activity occurred, (3) tobacco industry tactics, arguments or issues covered, and (4) anything else that reflects a potential theme. Avoid too generic tags (eg, 'industry interference'). Examples of suitable tags: 'Singapore,' 'menthol,' 'youth marketing,' 'tobacco farming,' 'trade treaties.'

For each reference, follow this standardized format:

{unformatted EndNote reference}

Tag 1; tag 2; tag 3; tag 4; etc

Outline of the paper

- Info piece #1
- Info piece #2

Double-checking

1. A second researcher double-checks the recorded data and tags against the original sources.
2. Where there are discrepancies, the two researchers resolve via discussion. If there is still a discrepancy, a third researcher is asked to check and the discrepancy is resolved via discussion between the three researchers.

Coding

3. The researcher who did the analysis reads through their data again to put together a list of their tags, grouping similar ones together into 'themes' (eg, 'youth marketing' and 'targeting kids' might become a single theme) and putting related themes into

categories of themes (eg, ‘youth marketing’ and ‘targeting females’ might become a category on tobacco marketing).

4. The researchers compare their themes and, in discussion, decide on the final set of themes and categories.
5. They go back into their data and reassign new tags based on the finalized themes.
6. A second researcher double-checks the recorded data and tags against the original sources.
7. Where there are discrepancies, the two researchers resolve via discussion. If there is still a discrepancy, a third researcher is asked to check and the discrepancy is resolved via discussion between the three researchers.
